# Deepening responses associated with improved progression-free survival with ixazomib versus placebo as posttransplant maintenance in multiple myeloma

**DOI:** 10.1038/s41375-020-0819-8

**Published:** 2020-04-23

**Authors:** Hartmut Goldschmidt, Meletios A. Dimopoulos, S. Vincent Rajkumar, Katja C. Weisel, Philippe Moreau, Wee-Joo Chng, Gábor Mikala, Michele Cavo, Karthik Ramasamy, Kaveri Suryanarayan, Zhaoyang Teng, Richard Labotka, Maria Victoria Mateos

**Affiliations:** 1 Internal Medicine V and National Center for Tumor Diseases (NCT), University Clinic Heidelberg, Heidelberg, Germany; 2 Hematology & Medical Oncology, Department of Clinical Therapeutics, National and Kapodistrian University of Athens, School of Medicine, Athens, Greece; 3 Division of Hematology, Department of Internal Medicine, Mayo Clinic, Rochester, MN USA; 4 Department of Oncology, Hematology and Bone Marrow Transplantation with Section of Pneumology, University Medical Center Hamburg-Eppendorf, Hamburg, Germany; 5 Department of Hematology, University Hospital Hôtel Dieu, University of Nantes, Nantes, France; 6 Department of Haematology-Oncology, National University Cancer Institute, National University Health System, Singapore, Singapore; 7 Cancer Science Institute of Singapore, National University of Singapore, Singapore, Singapore; 8 South Pest Central Hospital, National Institute for Hematology and Infectious Diseases, Budapest, Hungary; 9 Seràgnoli Institute of Hematology, University of Bologna, Bologna, Italy; 10 Oxford University Hospitals, NHS Foundation Trust, Oxford Myeloma Centre for Translational Research, Oxford, UK; 11 Millennium Pharmaceuticals, Inc., a wholly owned subsidiary of Takeda Pharmaceutical Company Limited, Cambridge, MA USA; 12 Hematology, Hospital Universitario de Salamanca, University Hospital of Salamanca, IBSAL, CIC, IBMCC (USAL-CSIC), Salamanca, Spain; 13 Present Address: Servier Pharmaceuticals, Boston, MA USA

**Keywords:** Myeloma, Randomized controlled trials

## Abstract

In the TOURMALINE-MM3 study, post-autologous stem cell transplantation maintenance therapy with the oral proteasome inhibitor ixazomib versus placebo significantly improved progression-free survival (PFS), with a favorable safety profile. With ixazomib versus placebo maintenance, deepening responses occurred in 139/302 (46%) versus 60/187 (32%) patients with very good partial response or partial response (VGPR/PR) at study entry (relative risk 1.41, *P* = 0.004), and median time to best confirmed deepened response was 19.9 versus 30.8 months (24-month rate: 54.2 versus 41.4%; hazard ratio (HR): 1.384; *P* = 0.0342). Median PFS in patients with VGPR/PR at study entry was 26.2 versus 18.5 months (HR: 0.636, *P* < 0.001) with ixazomib versus placebo; in a pooled analysis across arms, in patients with versus without deepening responses, the median PFS was not reached versus 15.9 months (HR: 0.245, *P* < 0.001). In patients with deepening responses, 24-month PFS rate was 77.4 versus 68.3% with ixazomib versus placebo (HR: 0.831; *P* = 0.466); in patients without deepening responses, median PFS was 17.9 versus 14.1 months (HR: 0.741; *P* = 0.028). These analyses demonstrate the significantly higher rate of deepening responses with ixazomib versus placebo maintenance and the association between deepening response and prolonged PFS.

## Introduction

Depth of response in multiple myeloma (MM) is an important prognostic factor that is strongly associated with progression-free survival (PFS) and overall survival (OS). Specifically, deepening response has been correlated to prolonged PFS and OS [1, 2], including in the post-autologous stem cell transplantation (ASCT) maintenance therapy setting [3, 4], confirming the prognostic importance of continued sensitivity to treatment and ongoing elimination of the MM disease burden.

Post-ASCT maintenance therapy is becoming widely used in the treatment of patients with newly diagnosed MM [5]. Lenalidomide is the only agent currently approved in this setting [6], having demonstrated significant PFS and OS benefits in phase 3 clinical trials [7–11] and in a meta-analysis [12]. Data have shown that lenalidomide treatment results in deepening responses in a proportion of patients who commence maintenance therapy having achieved less than a complete response (CR) following ASCT. In the phase 3, IFM 2005-02 trial of lenalidomide versus placebo the rate of CR plus very good partial response (VGPR) was increased by 23 versus 17 percentage points during lenalidomide maintenance versus placebo, which also resulted in a significant improvement in PFS versus placebo [7]. Similarly, in the recently reported Myeloma XI trial, post-ASCT maintenance with lenalidomide versus observation resulted in a significantly prolonged PFS (hazard ratio (HR): 0.48; median 57 versus 30 months) [9], and preliminary data presented on the rates of deepening response showed improved responses (to CR/VGPR) in 15.8 versus 11.0% of patients [13].

There is a need for additional treatment options as post-ASCT maintenance therapy, as MM is a heterogeneous disease [14] that may have differential sensitivity to therapeutic classes of agents with different modes of action. Moreover, MM patients have different disease characteristics that may make the use of a particular drug or class of agent more appropriate than another; additionally, long-term use of some agents may be limited by their parenteral route of administration. The Dutch–Belgian Cooperative Trial Group for Hematology Oncology-65/German-speaking Myeloma Multicenter Group-HD4 phase 3 trial compared bortezomib–doxorubicin–dexamethasone (PAD) induction and bortezomib maintenance after high-dose melphalan and ASCT with vincristine–doxorubicin–dexamethasone (VAD) induction and thalidomide maintenance post-ASCT in MM patients aged 18–65 years. After a median follow-up of 96 months, PFS (with censoring at allogeneic transplantation) was significantly prolonged in the PAD-bortezomib versus VAD-thalidomide arm. The negative prognostic effects of deletion 17p13 and renal impairment at baseline on PFS and OS were partially abrogated in the PAD-bortezomib but not in the VAD-thalidomide arm [15, 16]. However, in the absence of a second randomization, the study was not able to isolate the maintenance effect of bortezomib versus thalidomide, and there remained the need for a proof-of-concept randomized controlled study of a proteasome inhibitor as post-ASCT maintenance.

The recently reported phase 3, double-blind, placebo-controlled TOURMALINE-MM3 study (NCT02181413) investigated the use of the oral proteasome inhibitor ixazomib versus placebo as post-ASCT maintenance therapy in newly diagnosed MM patients [17], the first such comparative study of a proteasome inhibitor in this setting. The primary endpoint was PFS and the trial demonstrated a 39% improvement in PFS with ixazomib versus placebo (median 26.5 versus 21.3 months; HR: 0.72, 95% confidence interval (CI): 0.58–0.89; *P* = 0.0023), with a favorable safety profile for ixazomib in this setting [17]. Response rates, including improvement and durability of response, were among the secondary endpoints of the study. Here we report comprehensive analyses on improvements seen in depth of response over the course of the study and the impact of these deepening responses on outcomes in TOURMALINE-MM3.

## Subjects and methods

The patient eligibility criteria and study design of the international, multicenter, double-blind, placebo-controlled, phase 3 TOURMALINE-MM3 study have been reported previously [17]. Briefly, NDMM patients with adequate hematologic, hepatic, and renal function who had received standard-of-care induction therapy followed by a single ASCT and who had achieved at least a partial response (≥PR) were randomized in a 3:2 ratio to receive ixazomib (*N* = 395) or matching placebo (*N* = 261) on days 1, 8, and 15 of 28-day cycles for up to 2 years or until progressive disease or unacceptable toxicity. The ixazomib dose was 3 mg in cycles 1–4 and 4 mg from cycle 5 if tolerated in cycles 1–4. Randomization was stratified by three factors: induction regimen (regimen containing a proteasome inhibitor but not an immunomodulatory drug versus regimen containing an immunomodulatory drug but not a proteasome inhibitor versus regimen containing both an immunomodulatory drug and a proteasome inhibitor); International Staging System (ISS) disease stage prior to induction (stage I versus stage II or III); and post-ASCT response (CR or VGPR versus PR). All patients provided written informed consent.

Patients were assessed for response and disease progression according to the International Myeloma Working Group 2011 criteria [18], with responses evaluated by an independent review committee (IRC). Assessments were conducted every treatment cycle and then every 4 weeks until disease progression. Minimal residual disease (MRD) was assessed in bone marrow aspirate samples collected at screening, as well as after 13 and 26 cycles, by eight-color flow cytometry with a sensitivity of 10^−5^.

The primary endpoint of TOURMALINE-MM3 was PFS per IRC assessment. The secondary endpoints included the rate of response improvement among patients who had a response of VGPR or PR at study entry. The aims of the present analyses were to evaluate the rates and timing of response improvement during ixazomib versus placebo maintenance and the impact of deepening of response on PFS. Rates of deepening of response were determined for the ixazomib and placebo groups, and a relative risk (including 95% CI) for achieving deepening of response was calculated; rates of deepening of response were compared between groups using a Cochran–Mantel–Haenszel test stratified by response at study entry. Rates of deepening of response were similarly compared between patients who were MRD-negative versus MRD-positive at study entry in a pooled analysis across arms.

PFS distributions were estimated using Kaplan–Meier methodology, with events defined as disease progression or death. Patients without events were censored at the date of last response assessment. A Cox proportional-hazard regression model stratified per the randomization stratification factors was used to determine HRs and 95% CIs for comparisons of PFS between patient groups. *P* values were determined based on stratified log-rank tests for comparisons between subgroups defined by treatment arm, and a non-stratified log-rank test for the comparison between patients with and without deepening responses, regardless of treatment arm. Time to best deepened response was similarly evaluated and compared between groups, with the *P* value determined based on a non-stratified log-rank test, and events defined as achievement of best deepened response; patients without events were censored at the date of first documentation of progressive disease, or the last response assessment that was stable disease or better.

## Results

### Patients

Patient demographics and disease characteristics were well-balanced between the ixazomib and placebo arms in the TOURMALINE-MM3 study (Table 1) [17]. Overall, 29% of patients had ISS stage III at diagnosis, 89% of patients had received induction therapy containing a proteasome inhibitor, including 30% who received a proteasome inhibitor plus an immunomodulatory drug, and 15% and 21% of patients in the ixazomib and placebo arms, respectively, had high-risk cytogenetics at diagnosis, defined as the presence of del(17p), *t*(4;14), and/or *t*(14;16). Per IRC assessment, at study entry 60 (15%), 213 (54%), and 89 (23%) patients in the ixazomib arm had a response of CR, VGPR, and PR, respectively, compared with 54 (21%), 152 (58%), and 35 (13%) patients in the placebo arm. At the time of this analysis, median follow-up was 30.9 months in the ixazomib arm and 31.3 months in the placebo arm. Patients in the ixazomib arm received a median of 25 cycles (range 1–26) of treatment and patients in the placebo arm received a median of 22 cycles (range 1–26).

**Table 1:** Patient and disease characteristics at study entry by treatment arm.

	Ixazomib *N* = 395	Placebo *N* = 261
Median age, years (range)	58 (24–73)	60 (37–73)
Male, *n* (%)	252 (64)	162 (62)
Race—White/Asian,^a^ *n* (%)	315 (80)/59 (15)	213 (82)/36 (14)
ISS stage I/II/III at diagnosis, *n* (%)	151 (38)/129 (33)/115 (29)	94 (36)/92 (35)/75 (29)
High-risk cytogenetics, *n* (%)^b^	61 (15)	54 (21)
Induction therapy (stratification factor), *n* (%)		
PI-containing [with/without immunomodulatory drug]	352 (89) [118 (30)/234 (59)]	233 (89) [78 (30)/155 (59)]
Immunomodulatory drug but no PI	43 (11)	28 (11)

*IRC* independent review committee, *ISS* International Staging System, *MRD* minimal residual disease, *PI* proteasome inhibitor.

^a^In addition, race was reported as Black or African American in 7 (2%) patients in the ixazomib arm and in 3 (1%) patients in the placebo arm; 14 (4%) and 9 (3%) patients, respectively, were of other race or did not have race reported.

^b^*t*(4;14), *t*(14;16), and/or del(17p).

### PFS in patients with CR, VGPR, or PR at study entry

**Fig. 1:**
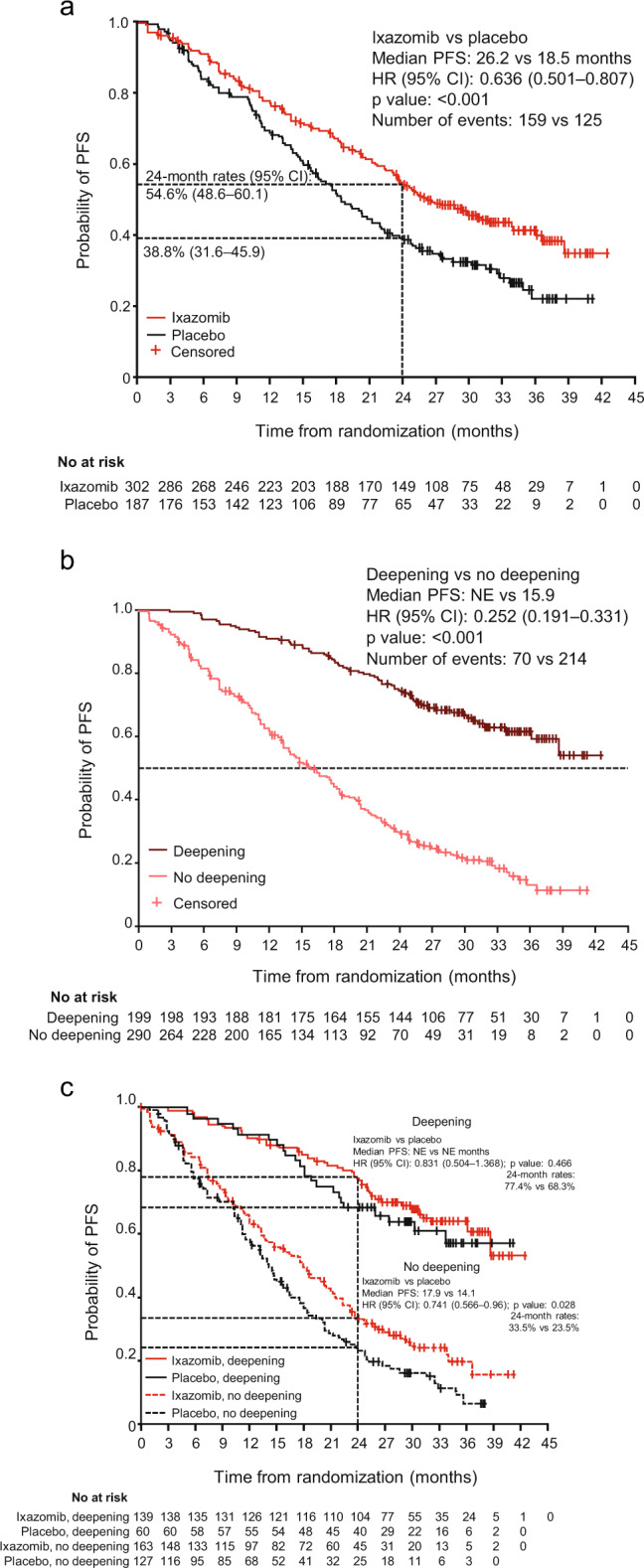
Kaplan–Meier estimates of progression-free survival (PFS). **a** All patients with a response of very good partial response (VGPR) or partial response (PR) at study entry, by treatment arm; **b** patients with VGPR or PR at study entry who did or did not achieve a confirmed deepening of response during or following study treatment, regardless of treatment arm; and **c** patients with VGPR or PR at study entry who did or did not achieve a confirmed deepening of response during or following study treatment, by treatment arm. Response and progression are as assessed by the independent review committee. *CI* confidence interval, *HR* hazard ratio, *NE* not estimable.

PFS benefit with ixazomib versus placebo according to the response at study entry has been reported previously [17]; in patients with CR, VGPR, or PR (based on patient-level data) at study entry, median PFS was 36.1 versus 25.8 months (HR: 0.88), 27.4 versus 21.3 months (HR: 0.69), and 23.5 versus 13.9 months (HR: 0.69), respectively. For the present analyses of PFS in patients with or without deepening response, PFS was evaluated by Kaplan–Meier analysis in 302 and 187 patients in the ixazomib and placebo arms who had a response of VGPR or PR at study entry per IRC assessment. PFS among these patients overall was longer in the ixazomib versus the placebo group (Fig. 1a); median PFS was 26.2 versus 18.5 months (HR: 0.636, *P* < 0.001), and 24-month PFS rates were 54.6% (95% CI: 48.6, 60.1) versus 38.8% (95% CI: 31.6, 45.9).

### Rates of deepening of response

Deepening of response was evaluated in 302 and 187 patients in the ixazomib and placebo arms who had a response of VGPR or PR at study entry per IRC assessment. At data cut-off, among these patients, confirmed deepening of responses (i.e., deeper response category confirmed by two consecutive assessments) were seen in 139 (46%) patients in the ixazomib group versus 60 (32%) patients in the placebo group (relative risk: 1.41, *P* = 0.004); overall- and individual-specific rates of deepening responses are summarized in Table 2.

**Table 2:** Summary of deepening responses among patients with a response of very good partial response (VGPR) or partial response (PR) at study entry, by treatment arm, as assessed by the independent review committee.

	Ixazomib, *n*/*N* (%)	Placebo, *n*/*N* (%)	Relative risk (95% CI)	*P* value
Patients who deepened to CR/patients in VGPR at study entry	92/213 (43)	48/152 (32)	1.368 (1.034, 1.810)	0.025
Patients who deepened to VGPR or better/patients in PR at study entry	47/89 (53)	12/35 (34)	1.540 (0.935, 2.537)	0.063
Patients with any deepening response/patients in VGPR or PR at study entry	139/302 (46)	60/187 (32)	1.407 (1.102, 1.797)	0.004

*P* values determined using a Cochran–Mantel–Haenszel test stratified by response at study entry.

*CI* confidence interval, *CR* complete response, *PR* partial response, *VGPR* very good partial response.

### PFS by deepening of response

PFS was analyzed according to whether or not patients who entered the study at VGPR or PR achieved a confirmed deepening of response during or following study treatment. A pooled analysis demonstrated that PFS was prolonged among those who had deepening of responses versus those who had no improvement in their response, regardless of treatment arm (Fig. 1b); median PFS was not reached versus 15.9 months (HR: 0.252, *P* < 0.001).

Analysis by treatment arm and by deepening of response showed that PFS was prolonged in patients who had confirmed deepening of responses versus those with no improvement in each arm individually (Fig. 1c). In patients who had deepening of responses, the median PFS was not reached in either the ixazomib or placebo group, with 24-month PFS rates of 77.4% (95% CI: 69.4, 83.5) and 68.3% (95% CI: 55.0, 78.5), respectively; the HR for the difference in PFS between the groups was 0.831 (95% CI: 0.504, 1.368; *P* = 0.466) in favor of ixazomib. In patients who did not have deepening of responses, the median PFS was 17.9 versus 14.1 months with ixazomib versus placebo, with 24-month rates of 33.5% (95% CI: 26.0, 41.2) and 23.5% (95% CI: 16.1, 31.6), respectively; the HR for PFS was 0.741 (*P* = 0.028) in favor of ixazomib.

### Kinetics and factors predictive of deepening of response

**Fig. 2:**
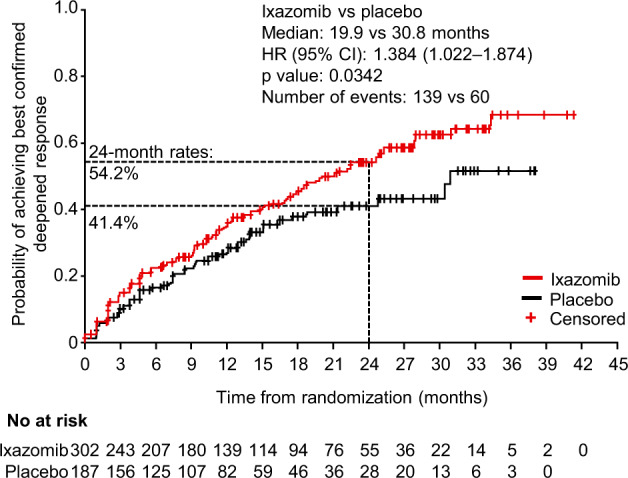
Kaplan–Meier estimate of time to best confirmed deepened response from randomization among patients with a response of very good partial response (VGPR) or partial response (PR) at study entry, by treatment arm, as assessed by the independent review committee. Patients who did not achieve a deepening response are censored at the date of first documentation of progressive disease, or the last response assessment that was stable disease or better. *CI* confidence interval, *HR* hazard ratio.

Time to best confirmed deepened response was evaluated by Kaplan–Meier analysis in 302 and 187 patients in the ixazomib and placebo arms who had a response of VGPR or PR at study entry (Fig. 2). Median time to best confirmed deepened response was shorter with ixazomib versus placebo (19.9 versus 30.8 months), and 24-month rates of deepening of response were 54.2% versus 41.4%, respectively. The HR for the comparison between groups was 1.384 in favor of ixazomib, indicating that there was a 38.4% improvement in the chance of deepening of response over time with ixazomib versus placebo (*P* = 0.0342).

**Fig. 3:**
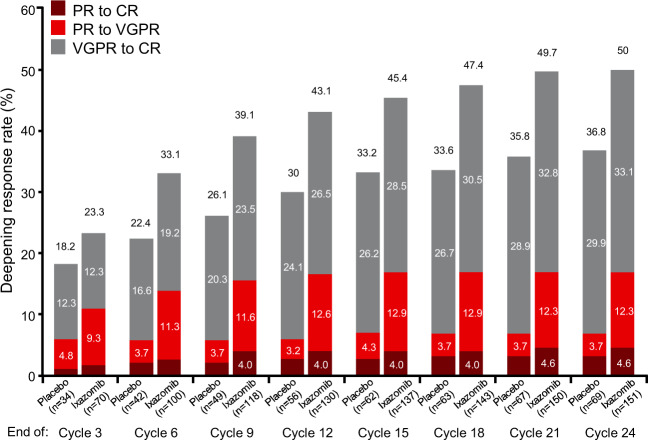
Cumulative rates of confirmed or unconfirmed deepening response over time, by treatment arm. The figure shows the proportions of patients with a response of very good partial response (VGPR) or partial response (PR) at study entry who had achieved a confirmed or unconfirmed deepening of response, overall and by degree of deepening of response, by the end of every 3 cycles of treatment. Numbers of patients with a deepening of response in each arm at each time point are shown on the x-axis.

Cumulative rates of confirmed or unconfirmed (i.e., a single response assessment showing a deeper response category) deepening of response over time are shown in Fig. 3 at 3-month intervals over the protocol-specified 2-year treatment period. At 12 months, 80 patients (27%) in the ixazomib arm had a deepening of response from VGPR to CR, compared with 45 patients (24%) in the placebo arm; similarly, 50 patients (17%) in the ixazomib arm had a deepened response from PR to CR or VGPR, compared with 11 (6%) in the placebo arm. Additional deepening of responses were recorded in both groups beyond 12 months; a further 20 (total: 100 [33%]) and 11 (total: 56 [30%]) VGPR patients on the ixazomib and placebo arms, respectively, had deepening of responses to CR after this time point (with 8 and 6 achieving response improvements beyond 18 months), and a further 1 and 2 PR patients, respectively, had deepening of responses to CR or VGPR. Reflecting the overall rates of confirmed deepening of responses, the overall proportion of patients with deepening of response (confirmed or unconfirmed) was higher in the ixazomib group versus the placebo group at each time point.

In addition to treatment arm, baseline MRD status was shown to be a strong predictor of deepening of response among patients who had a VGPR or PR at study entry, with an overall relative risk of achieving a deepening of response of 2.066 (*P* < 0.001) in favor of patients who were MRD-negative at study entry compared with those who were MRD-positive. Overall and individual specific rates of deepening of responses according to MRD status are summarized in Table 3; 63% and 32% of patients who were MRD-negative or MRD-positive at study entry, respectively, demonstrated deepening of responses.

**Table 3:** Summary of deepening responses among patients with a response of very good partial response (VGPR) or partial response (PR) at study entry, as assessed by the independent review committee, according to minimal residual disease (MRD) status at study entry.

	MRD-negative *N* = 124^a^ *n* (%)	MRD-positive *N* = 293^a^ *n* (%)	Relative risk (95%CI)	*P* value
VGPR at study entry	109 (88)	206 (70)		
Deepened to CR	66 (61)	54 (26)	2.310 (1.755, 3.040)	–
PR at study entry	15 (12)	87 (30)		
Deepened to VGPR or better	12 (80)	40 (46)	1.740 (1.238, 2.446)	
VGPR or PR at study entry	124 (100)	293 (100)		–
Deepening response	78 (63)	94 (32)	2.066 (1.668, 2.558)	<0.001

*P* value determined using a Cochran–Mantel–Haenszel test stratified by response at study entry.

*CI* confidence interval, *CR* complete response, *MRD* minimal residual disease, *PR* partial response, *VGPR* very good partial response.

^a^MRD status at study entry was evaluable in 357 and 228 patients in the ixazomib and placebo arms, respectively; 117 (33%) and 75 (33%), respectively, were MRD-negative, of whom 124 overall had a response of VGPR or PR at study entry; 225 (63%) and 139 (61%), respectively, were MRD-positive, of whom 293 overall had a response of VGPR or PR at study entry. MRD status was evaluated by eight-color flow cytometry with a sensitivity of 10^−^^5^.

## Discussion

The results of these analyses of the TOURMALINE-MM3 phase 3 study demonstrate that the achievement of deepening of response during post-ASCT maintenance was associated with prolonged PFS versus no response improvement. Post-ASCT maintenance with ixazomib resulted in a significantly higher rate of deepening of response compared with placebo indicating that the significant overall PFS benefit demonstrated with ixazomib versus placebo in TOURMALINE-MM3 [17] may be partly driven by enhanced ongoing anti-myeloma activity in a higher proportion of patients in the ixazomib arm. The HR for the difference in PFS between ixazomib and placebo patients who had deepening of response was 0.831 (95% CI: 0.504, 1.368), in favor of ixazomib. This limited benefit may be due to the fixed duration of ixazomib maintenance (~2 years) used in the study; in these favorable-prognosis patients with longer PFS, the emerging benefit seen at 24 months (Fig. 1c) may have been sustained or enhanced through the use of continued ixazomib maintenance. Prolonged follow-up would be needed to confirm whether a more substantial effect was seen subsequent to achieving maximal response depth in patients who had had a deepening response. However, ixazomib did result in a more substantial improvement in PFS compared with placebo in patients who entered the study in VGPR or PR and saw no improvement in their depth of response (HR: 0.741; 95% CI: 0.566, 0.96). Taken together, these results suggest that the overall PFS benefit with ixazomib versus placebo is driven through both types of patients. A substantial proportion of patients harbor tumors that are clinically sensitive to ongoing cytoreduction with ixazomib, resulting in an enhanced rate of deepening of response, which correlates to prolonged PFS (acknowledging the potential for immortal time bias associated with time taken to achieve improvement in response). In addition, patients who do not demonstrate deepening of response nevertheless benefit from sensitivity to ixazomib in terms of disease control/stabilization, as evidenced by the prolonged maintenance of existing response compared with placebo.

The association of depth of response with improved outcomes is well-established [19–22] and is supported by the data from TOURMALINE-MM3. In addition, our results showing superior PFS in patients achieving deepening of responses during maintenance are supported by data from other studies, including two separate retrospective analyses that demonstrated both PFS and OS improvements in patients who had a deepening response post-ASCT [3, 4]. More broadly, a recent analysis has shown that patients who take longer to achieve their “plateau” of best response have longer PFS and OS than those who achieve maximal response more rapidly [2]. Similar findings were reported from an analysis of response kinetics and outcomes data from the TOURMALINE-MM1 phase 3 trial of ixazomib–lenalidomide–dexamethasone versus placebo–lenalidomide–dexamethasone in relapsed/refractory MM, in which PFS was longer among so-called “late responders” versus those who achieved best response to treatment after a shorter period of time [23].

Response improvements are to be expected regardless of treatment arm in patients posttransplant, due to the continued decline of the plasma cell clone and ongoing clearance of the M-protein seen post-ASCT specifically in those patients with sensitive, less proliferative, and more mature clones, as well as those with immunoglobulin isotypes with longer half-lives, e.g., IgG [3]. In addition, such response improvements may be associated with improved post-ASCT T cell reconstitution and the development of prognostically favorable immune signatures that offer prolonged anti-MM immunological surveillance [24–26]. The rate of “delayed” deepening of responses post-ASCT in the placebo group in TOURMALINE-MM3 was 32%, with patients entering the study a median of 3.4 months after ASCT [17]. This is similar to the rate reported for the Mayo Clinic series by Gonsalves et al., in which 39% of patients achieved a continued response post day 100 without additional therapy, 67% of whom had a deeper response per IMWG criteria [3]. In contrast, preliminary data from the Myeloma XI trial presented at ASH 2017 indicated an 11% rate of response improvement among patients undergoing only observation post-ASCT [13]. These discrepant results could have been caused by different methodologies, i.e., in melphalan conditioning regimen prior to ASCT and/or induction therapy. Nevertheless, the evidence suggest that delayed response occurs with a limited number of instances of deepening response occurring more than 2 years after ASCT in the placebo arm of the present study and the observation arm of Myeloma XI [13]. One hypothesis is that such late effects may be associated with the favorable immune signatures discussed above or may be due to fluctuations in M-protein levels in patients with levels close to the response category thresholds. Further investigation is ongoing to understand these observations.

In this context, 46% rate of deepening responses seen with ixazomib maintenance in 139 patients in TOURMALINE-MM3 is notable and suggests a potential synergy in some patients between the mechanism of anti-myeloma action of ixazomib and the post-ASCT immunologic disease control mechanisms discussed in the previous paragraph. Preliminary data from Myeloma XI on response improvement with lenalidomide post-ASCT maintenance indicated a rate of 15.8 versus 11.0% with no maintenance [13], in the context of a median PFS of 57 versus 30 months [9], and results from the IFM 2005-02 trial of lenalidomide versus placebo as post-ASCT maintenance showed an increase in the rate of CR/VGPR of 23 versus 17 percentage points during maintenance, with a median PFS of 41 versus 23 months [7]. Differences in rates between studies/analyses must be interpreted with caution due to potential differences in response assessment rigor and frequency and due to differences in prior induction therapy, as well as differences in the depth of response achieved following prior induction therapy and post-ASCT. However, the data collectively suggest that the PFS benefit of maintenance therapy arises both from ongoing disease control among patients who have reached maximal response and from increasing depth of response among some patients, recognizing that a proportion of the latter effect may be associated with ongoing disease elimination post-ASCT. Thus, in routine practice, sequential analyses of response data for patients commencing maintenance therapy in PR or VGPR may be valuable in identifying an ongoing deepening response, thereby providing support for continuing with maintenance therapy, with the goal of achieving CR and a prolonged PFS.

It must be acknowledged that lenalidomide results in a greater differential median PFS benefit versus placebo than ixazomib [7, 9, 12, 17]. However, data from these trials do not enable the head-to-head comparison of two different drugs with different mechanisms of action, which may provide differential activity in terms of response and outcome in an individual patient. The subset of patients identified in our study who had deepened responses, presumably due to clinical sensitivity for ongoing cytoreduction with ixazomib maintenance, benefit from a prolonged PFS that appears comparable to that seen with lenalidomide in the Myeloma XI trial [9]. In the context of the agents’ different risk–benefit profiles [17], and acknowledging the overall difference in PFS benefit, each agent may provide different benefit in different subsets of patients, as discussed in the primary report of TOURMALINE-MM3 [17] and as also suggested in the presentation of preliminary findings of a randomized phase 2 head-to-head trial of ixazomib versus lenalidomide maintenance after ixazomib–lenalidomide–dexamethasone consolidation [27].

Our findings should be considered in the context of the strengths and limitations of the TOURMALINE-MM3 study. This was a rigorously conducted, double-blind, placebo-controlled study that clearly established the activity of ixazomib as post-ASCT maintenance therapy; however, due to it being the standard-of-care treatment at the time the study was initiated, the comparator arm was placebo rather than the now-established standard of care, lenalidomide [17]. The different types and durations of induction therapy may have resulted in differential sensitivity to proteasome inhibitor maintenance with ixazomib, although prior induction therapies were well-balanced between arms [17]. Outcomes may have been influenced by timing of starting treatment within the 40-day window, between day 75 and day 115 post-ASCT, during which patients could be screened and randomized, and during which changes in response status might have occurred prior to randomization [17]. Finally, it is feasible that the use of the initial lower dose of ixazomib (3.0 mg) prior to dose escalation to 4.0 mg from cycle 5 onwards if tolerated [17], and the fixed duration of maintenance of 2 years, may have impacted the degree of response and PFS benefit demonstrated.

In conclusion, these analyses from the TOURMALINE-MM3 phase 3 study of ixazomib versus placebo as post-ASCT maintenance demonstrated that, overall, patients who achieved deepening of response had substantially longer PFS than those who did not, and ixazomib maintenance resulted in a significantly higher rate of deepening responses than placebo. Furthermore, ixazomib also benefited patients with VGPR or PR at study entry who did not achieve a deepening response, resulting in longer PFS versus placebo, an important finding in the context of a recent analysis demonstrating the long-term survival benefit of sustaining any level of response post-ASCT [28]. These dual benefits of ixazomib maintenance warrant further investigation to determine whether differential benefits are provided in patients with different residual clonal disease, thereby potentially supporting the rationale for an ixazomib-based combination maintenance approach in some patients.
